# Unraveling the Enigma of Post-sleeve Differential Liquid Tolerance: A Time-Resolved MRI Study

**DOI:** 10.1007/s11695-025-08276-8

**Published:** 2025-10-12

**Authors:** Athar Elward, Ahmad Omar, Ahmed Abdelsalam, Islam Shawali, Mina Makram, Ahmed Refaat

**Affiliations:** 1 Department of general surgery, Cairo University, Giza, Egypt; 2 Department of diagnostic radiology, Cairo University, Giza, Egypt

**Keywords:** Sleeve gastrectomy, Liquids tolerance, Dynamic MRI

## Abstract

**Background:**

Laparoscopic sleeve gastrectomy is one of the restrictive bariatric procedures that often impair food and fluid tolerance and negatively impact quality of life as it affects patients’ weight loss, hospital stay, and lifestyle.

**Aim:**

The objective of this study was to evaluate water and juice tolerance after LSG using dynamic magnetic resonance imaging (MRI) of the stomach to determine the relationship between fluid tolerance and gastric motility.

**Methods:**

The prospective cohort study enrolled 17 adult subjects of both sexes, with a body mass index (BMI) greater than 40 kg/m^2^ without comorbidities or 35 kg/m^2^ with comorbidities, who underwent LSG. Patients were monitored for water and juice tolerance, and dynamic MRI imaging for gastric motility patterns was obtained at one and six months postoperatively.

**Results:**

More than half of participants (58.8%) reported difficulty tolerating water, while 5.9% of participants expressed early difficulty tolerating juice. After six months, water tolerance improved significantly. Compared to early dynamic MRI, late MRI gastric motility patterns revealed an improvement trend in the form of a significant decrease in antral frequency and an increase in antral velocity, sleeve transit, and contraction height for both water and juice (*p* < 0.001). Among patients with improved water tolerance (6 patients), late MRI findings were significantly improved compared to their early MRI findings (*p* < 0.001). Patients’ early wave contraction height was significantly different between water and juice at early and late imaging (*p* < 0.001).

**Conclusion:**

Time-resolved gastric MRI is a useful tool to explain the roots of post-LSG differential liquid tolerance. The gastric antrum is the only coordinated and functionally motile part of the gastric sleeve. An overall significantly improved motility pattern trend was achieved by the end of the study, with the increase in antral contraction height being the most consistent component of that trend. Water tolerance improves significantly 6 months after LSG but remains worse than juice tolerance.

## Introduction

Laparoscopic sleeve gastrectomy (LSG) is one of the restrictive bariatric procedures that often impair food and fluid tolerance and negatively impact quality of life. Meanwhile, weight loss, patient lifestyle, and safe hospital discharge all depend on good liquid tolerance, especially water [1].

Inadequate water intake may predispose patients to a hypercoagulable state, which in turn may cause venous thrombosis [2]. It has also been suggested that adequate water intake contributes to weight loss by increasing the metabolic pathway in the mitochondria [3]. Reduced hydration in patients may predispose them to cardiovascular disease, diabetes, and Alzheimer's disease in the long term [4]. Numerous studies have evaluated food and its various types of tolerance, but only a few have evaluated liquid tolerance after LSG, specifically water tolerance [5–7].

Magnetic resonance imaging (MRI) is well known for its ability to provide high contrast in soft tissues and for its potential to analyze complex motion patterns with high temporal resolution at the sub-second level [8, 9]. High temporal resolution imaging is made possible by the extremely short repetition periods of the balanced steady-state free precession sequence family [10]. For example, variations of the sequences are commonly used for MR defecography or cardiac imaging. Dynamic MRI was used to assess gastric motility and emptying in healthy individuals [11, 12]. However, the studies evaluating gastric motility and emptying in adults after LSG are scarce [9]. This study aimed to evaluate water and juice tolerance after LSG to elucidate the possible causes, the relationship between water and juice tolerance and gastric contractility variations, and to evaluate excess weight loss as the primary goal of any bariatric surgery.

## Methods

### Ethical Considerations

After approval by the research ethics committee of Faculty of Medicine, Cairo University, (approval number: MD-333-2022), all study participants provided informed written consent. The confidentiality of each participant’s information was guaranteed.

### Study Design, Setting, and Location

This prospective cohort study was conducted at the Faculty of medicine, Cairo University hospital, general surgery Unit, between September 2022 and March 2024.

### Eligibility Criteria

The study population comprised adults of both sexes between the ages of 18 and 60 years with a body mass index (BMI) greater than 40 kg/m^2^ without comorbidities or 35 kg/m^2^ with comorbidities, such as arthritis, obstructive sleep apnea, or hypertension, who were being treated with LSG. Patients who had previously undergone major abdominal surgery, intragastric balloon, hiatal hernia, postoperative leakage or bleeding, autoimmune disease in addition to pregnancy, steroid treatment, heart disease, or hypothyroidism were excluded from the study. Furthermore, patients diagnosed with diabetes mellitus with HbA1c greater than 6.5 mg/dL or who were not suitable for MRI, such as the presence of a pacemaker, claustrophobia, and body weight over 120 kg, were excluded from the study.

### Preoperative Assessment

A comprehensive evaluation was performed to assess the patients' general health, comorbidities, risk factors, psychological status, and ability to adhere to a postoperative protocol. Laboratory and radiographic studies were performed, including dynamic MRI. In addition, endocrine and cardiovascular evaluations were performed, including pulmonary function testing, electrocardiography, and echocardiography. Supplementary tests for existing comorbidities were performed as needed. Patients received respiratory physiotherapy and were advised to be physically active. All patients received antithrombotic and antibacterial prophylaxis.

### Operative Details

Laparoscopic sleeve gastrectomy was performed by the same surgeon under general anesthesia. The procedure was carried out by dissecting the greater curvature, commencing at approximately 5–6 cm from the pylorus, utilizing a 36 Fr. gastric tube for calibrating the sleeve [13]. Methylene blue testing was performed in all cases to verify the integrity of the stapler line.

Postoperative mobilization was encouraged a few hours after the procedure. On the second postoperative day, patients were permitted to resume oral fluid intake. A post-surgical diet progression from liquids to solids over 6 to 8 weeks was advised for all patients, with a focus on meeting protein and fluid requirements, with additional daily micronutrient supplement intake.

### MRI and Liquid Tolerance Assessment

A total of two MRI exams were scheduled for each patient: a surgical post-op evaluation performed one month after surgery (early), and a follow-up examination conducted six months post-surgery (late). All examinations were carried out on a short-bore 1.5-T scanner (Optima MR450w GEM, GE Healthcare, United States) with a gantry diameter of 70 cm, a configuration that is particularly well-suited for obese patients. All participants fasted at least six hours before the examinations.

Patients were offered 50 cc of tap water at room temperature and instructed to consume as much as possible in an upright position within a maximum time period of five minutes. Following this, 50 cc of sugar-free juice at room temperature was offered with an interval of one hour between them. Patients were asked to grade their tolerance to water and juice separately as “tolerating,” “tolerating with difficulty” (where “difficulty” was defined as dysphagia or epigastric pain during drinking), or “not tolerating at all.” After the ingestion of water, patients were scanned once, and subsequently, after the ingestion of juice, they were scanned a second time.

The achieved ingestible volume was meticulously documented. Then, the patients were positioned in a supine position, with their heads positioned in a posterior direction on the scanner table. The signal reception was facilitated by the integration of spine coils within the scanner table and the placement of a body matrix coil on the upper abdomen. Static images of the upper abdomen were subsequently acquired using single-shot fast spin echo (SS-FSE) sequences in axial, coronal, and, if necessary, sagittal planes, encompassing the sleeve region. Subsequently, dynamic single-slice acquisitions employing cine balanced steady-state free precession (bSSFP) measurements were planned on these images in various orientations.

The slice position and orientation were selected based on the individual anatomy to ensure optimal depiction of the motility of the sleeve (sagittal or axial oblique) and the antrum (coronal oblique). The imaging parameters for the different sequences are outlined below: coronal and sagittal SS-FSE (TR, 1,010 ms; TE, 102 ms; slice thickness, 6 mm; slice gap, 1.2 mm; flip angle, 150°; matrix, 320 × 320), axial SS-FSE (TR, 800 ms; TE, 102 ms; slice thickness, 5 mm; slice gap, 0. The first sequence was acquired with the following parameters: 5 mm; flip angle, 150°; matrix, 512 × 408. The second sequence was acquired with the following parameters: single-slice cine bSSFP (TR, 4.62 ms; TE, 2.31; slice, 5 mm; flip angle, 40°; matrix, 192 × 180; time frames, 35; acquisition time, 30 s; temporal resolution, 0.86 s).

All sequences were obtained during a single breath-hold. The protocol initiated with bi- or triplanar SS-FSE imaging, followed by repetitive sets of cine acquisitions with varying angulations for dynamic analysis. These acquisitions continued until the stomach was completely emptied or the total examination time reached 30 min.

### MRI Analysis

Dynamic sequences were examined using cine loops for qualitative analysis of the gastric motility. The appearance, shape, and motion of peristaltic folds and the general gastric motility in the antrum and sleeve were meticulously documented.

To perform a quantitative analysis, the number of folds appearing in the antrum and the sleeve was recorded separately, and the fold frequency was calculated as the ratio between the number of folds observed and the total time the respective part of the stomach was depicted in the respective images (number of sequences multiplied by individual acquisition time).

The dimensions of antral folds were measured on para-coronal planes at the inferior aspect of the antrum when the folds reached their maximum height. The temporal dynamics of the antral folds were then determined by calculating the speed of these structures from the distance between their positions on two images from a single series. This distance was then divided by the number of frames between the key images, with the result multiplied by the temporal resolution of the images.

### Postoperative Care

Postoperative follow-up was performed during the first seven days postoperatively to assess the surgical site and remove sutures.

Subsequent dynamic MRI evaluations, including sleeve transit time, contraction wave height, velocity, and frequency of contraction waves, were performed at one and six months postoperatively.

Weight loss was assessed using BMI and excess weight loss percentage (EWL%). Measurements were taken before surgery and at six months postoperatively.

### Study Outcomes

The primary outcome of the study included the early and late subjective water and juice tolerance, early and late dynamic MRI motility patterns for water and juice, and correlation between early and late findings and between subjective and MRI objective findings. Secondary outcomes encompassed the patients’ demographics, with the early and late %EWL and their correlations with the primary outcomes.

### Sample Size

Based on data from previous, comparable research [9], and considering the use of dynamic MRI to identify sleeve motility patterns during early and late consumption of various fluids and comparing this to subjective tolerance of fluids in the early and late phases as the primary outcome, the sample size for this study was determined using the Epi Info Calculator. It assumes 80% power, a significance level of 0.05, and a 95% confidence interval. There were 17 subjects in the sample.

### Statistical Analysis

The data were subjected to statistical analysis using IBM SPSS software, version 21.0 (Armonk, NY: IBM Corp., released 2011). Categorical data were presented as numerical values and percentages. The normality of continuous data was evaluated using the Shapiro–Wilk test. Quantitative data were reported as range, mean, standard deviation, median, and interquartile range (IQR). Parametric tests used for quantitative normally distributed data were the independent *T* test and the ANOVA test; non-parametric statistical tests used for qualitative data were the chi-square test and the Mann–Whitney test for quantitative not normally distributed. Level of significance was considered if the *p* value was below 0.05.

## Results

A total of 17 patients were enrolled in the study. The mean age of the enrolled patients was 38.88 ± 8.59 years, with a predominance of females (82.4%). Only 15 patients completed the 6-month follow-up period, with two patients (1 female and 1 male) lost to follow-up after they had the early MRI examination done. The mean preoperative BMI was 45.11 ± 4.64 kg/m^2^. Regarding obesity-related comorbidities, 29.4% of the patients exhibited hypertension, while 5.9% had both hypertension and hepatitis C. Most of the participants (82.4%) had no prior surgical history. At the one (early)—and six-month (late) postoperative intervals, there was a significant increase in early and late % EWL (28.10 ± 8.77 and 67.99 ± 9.21) (Table 1).

**Table 1:** Patients’ baseline characteristics (*n* = 17)

		*n* (%)
Sex, *n* (%)	Male	3 (17.6%)
	Female	14 (82.4%)
Age, years	Min–Max	24–52
	Mean ± SD	38.88 ± 8.59
Height, cm	Min–Max	156–170
	Mean ± SD	162.18 ± 4.23
Initial weight, kg	Min–Max	83–145
	Mean ± SD	118.62 ± 13.86
BMI, kg/m^2^	Min–Max	33.70–53.30
	Mean ± SD	45.11 ± 4.64
Medical history, *n* (%)	Virus C & hypertension	1 (5.9%)
	Hypertension	5 (29.4%)
	Free	11 (64.7%)
Surgical history, *n* (%)	Hysterectomy	1 (5.9%)
	Disc prolapse	1 (5.9%)
	Appendectomy	1 (5.9%)
	Free	14 (82.4%)
Early excess weight loss, %	Min–Max	18.80–56.30
	Mean ± SD	28.10 ± 8.77
Late excess weight loss, %	Min–Max	52.80–84.00
	Mean ± SD	67.99 ± 9.21

*BMI* body mass index, *Max* maximum, *Min* minimum, *n* number, *SD* standard deviation

Ten patients (58.8%) exhibited early reduced water tolerance, while only 3 patients (20%) demonstrated late reduced water tolerance. In contrast, only one patient (5.9%) displayed early reduced juice tolerance, and no patients exhibited late reduced juice tolerance. There was a significant improvement in patients with reduced water tolerance at 6 months (*p* = 0.026) (Table 2). Early water tolerance was significantly worse than juice tolerance (*p* = 0.0012), while late water tolerance was nearly significantly worse than juice tolerance (*p* = 0.0726).

**Table 2:** Early and late tolerance for juice and water (*n* = 17)

		Early	Late	*p* value
Water tolerance	**Tolerating**	7 (41.2%)	12 (80%)	0.026 *
	**Tolerating with difficulty**	10 (58.8%)	3 (20%)	
Juice tolerance	**Tolerating**	16 (94.1%)	15 (100%)	
	**Tolerating with difficulty**	1 (5.9%)	0	

*n* number

*Significant at *p* < 0.05

The vertical part of the gastric sleeve was either immotile or exhibited haphazard, insignificant contractions during early and late imaging. Consequently, our focus was on documenting antral motility patterns only. Late MRI findings showed a highly significant decrease in antral frequency for both water (2.66 ± 0.40 vs 2.48 ± 0.39, *p* < 0.001) and juice (2.72 ± 0.38 vs 2.40 ± 0.37, *p* < 0.001), while there was a highly significant increase in antral velocity, sleeve transit time, and contraction height for both water and juice (*p* < 0.001) (Table 3).

**Table 3:** Correlation between early versus late water and early versus late juice dynamic MRI motility patterns (*n* = 17)

Dynamic MRI motility patterns		Early	Late	*p* value
Water	**Antral frequency/min**, mean ± SD	2.66 ± 0.40	2.48 ± 0.39	< 0.001*
	**Antral velocity**, mm/sec, mean ± SD	3.49 ± 0.69	3.81 ± 0.58	< 0.001*
	**Sleeve transit time**, min, mean ± SD	24.06 ± 3.90	27.47 ± 2.53	< 0.001*
	**Contraction height**, mm, mean ± SD	4.56 ± 0.70	5.20 ± 0.74	< 0.001*
Juice	**Antral frequency/min**, mean ± SD	2.72 ± 0.38	2.40 ± 0.37	< 0.001*
	**Antral velocity mm/sec**, mean ± SD	3.56 ± 0.71	3.86 ± 0.56	0.016 *
	**Sleeve transit time**, min, mean ± SD	23.59 ± 3.97	28.13 ± 2.72	< 0.001*
	**Contraction height**, mm, mean ± SD	4.98 ± 0.76	5.57 ± 0.76	< 0.001*

*Sec* second, *n* number, *SD* standard deviation

*Significant at *p* < 0.05

Six patients showed improved late water tolerance, and their MRI motility patterns revealed a significant decrease in water antral frequency (*p* = 0.041) and a significant increase in water antral velocity (*p* = 0.028), sleeve transient time (*p* = 0.002), and contraction height (*p* < 0.001) in late MRI findings compared to their early MRI findings (Table 4).

**Table 4:** Correlation between early and late dynamic MRI motility patterns for patients who showed improved water tolerance (*n* = 6)

Improved water tolerance, mean ± SD	Early	Late	*p* value
Water antral frequency/min	2.73 ± 0.41	2.53 ± 0.37	0.041*
Water antral velocity, mm/sec	3.68 ± 0.64	3.87 ± 0.65	0.028*
Water sleeve transit time, min	24.17 ± 1.83	27.00 ± 2.45	0.002*
Water contraction height, mm	4.45 ± 0.83	5.23 ± 0.83	< 0.001*

*n* number, *SD* standard deviation

*Significant at *p* < 0.05

While comparing early dynamic MRI motility patterns for water versus juice and late MRI patterns of water versus juice did not reveal significant differences except for contraction height, which was the only parameter showing a highly significant difference between them in both early and late phases (*p* < 0.001–*p* < 0.001) (Table 5).

**Table 5:** Correlation between early water versus early juice and late water versus late juice dynamic MRI motility patterns (*n* = 17)

MRI motility patterns		Water	Juice	*p* value
Early, mean ± SD	**Antral frequency/min**	2.66 ± 0.40	2.72 ± 0.38	0.166
	**Antral velocity mm/sec**	3.49 ± 0.69	3.56 ± 0.71	0.067
	**Sleeve transit time**, min	24.06 ± 3.90	23.59 ± 3.97	0.191
	**Contraction height**, mm	4.56 ± 0.70	4.98 ± 0.76	< 0.001*
Late, mean ± SD	**Antral frequency/min**	2.48 ± 0.39	2.40 ± 0.37	0.054
	**Antral velocity** mm/sec	3.81 ± 0.58	3.86 ± 0.56	0.164
	**Sleeve transit time**, min	27.47 ± 2.53	28.13 ± 2.72	0.065
	**Contraction height**, mm	5.20 ± 0.74	5.57 ± 0.76	< 0.001*

*sec* second, *n* number, *SD* standard deviation

*Significant at *p* < 0.05

Correlation between dynamic MRI motility patterns of patients tolerating water and those tolerating it with difficulty at early and late stages revealed no significant differences (Table 6).

**Table 6:** Correlation between the dynamic MRI motility patterns of patients tolerating water and those tolerating it with difficulty at early and late stages (*n* = 17)

MRI motility patterns of water, mean ± SD		Tolerating	Tolerating with difficulty	*p* value
Early	**Antral frequency/min**	2.70 ± 0.33	2.64 ± 0.46	0.772
	**Antral velocity, mm/sec**	3.43 ± 0.72	3.53 ± 0.70	0.775
	**Sleeve transit time, min**	23.29 ± 5.53	24.60 ± 2.41	0.511
	**Contraction height, mm**	4.47 ± 0.60	4.62 ± 0.78	0.679
Late	**Antral frequency/min**	2.51 ± 0.37	2.37 ± 0.57	0.596
	**Antral velocity, mm/sec**	3.84 ± 0.61	3.67 ± 0.47	0.655
	**Sleeve transit time, min**	27.50 ± 2.24	27.33 ± 4.16	0.923
	**Contraction height, mm**	5.10 ± 0.71	5.60 ± 0.87	0.312

*n* number, *SD* standard deviation

The %EWL of patients with reduced late water tolerance (60.90 ± 4.86) was lower than that of patients with good late water tolerance (69.77 ± 9.31), but without statistically significant differences. There were no significant differences between excess weight loss %, age, initial weight, height, and BMI among patients with different early and late water tolerance (Table 7).

**Table 7:** Correlation between excess weight loss %, age, initial weight, height, and BMI among patients with early and late water tolerance (*n* = 17)

Early water tolerance, mean ± SD	Tolerating	Tolerating with difficulty	*p* value
Early excess weight loss %	26.70 ± 6.21	29.08 ± 10.41	0.598
Age, years	37.57 ± 8.77	39.80 ± 8.80	0.614
Initial weight, kg	117.50 ± 8.31	119.40 ± 17.14	0.791
Height, cm	163.57 ± 3.99	161.20 ± 4.32	0.269
BMI	44.06 ± 3.33	45.85 ± 5.42	0.451
Late water tolerance, mean ± SD			
Late excess weight loss %	69.77 ± 9.31	60.90 ± 4.86	0.141
Age, years	38.08 ± 9.55	42.33 ± 8.02	0.493
Initial weight, kg	119.04 ± 9.30	126.67 ± 17.56	0.302
Height, cm	162.92 ± 4.34	161.00 ± 4.58	0.509
BMI	44.94 ± 3.48	48.73 ± 4.15	0.126

*n* number, *SD* standard deviation, BMI body mass index

## Discussion

The root causes of the phenomenon of post-LSG differential liquid tolerance are as yet a mystery. This study evaluated water and juice tolerance after LSG to elicit the possible causes, the relation between water and juice tolerance and gastric sleeve contractility variations, as well as to assess excess weight loss.

Our main findings included a significant difference in tolerance between water and juice after LSG. Nearly two-thirds of patients complained of decreased water tolerance one month after surgery. After six months, water tolerance improved significantly, and only one-fifth of the patients continued to suffer from reduced water tolerance. Compared to early dynamic MRI, late MRI gastric motility patterns findings revealed a significant decrease in water and juice antral frequency and an increase in water and juice antral velocity, sleeve transit, and contraction height. In addition, we did not find any significant differences in age, weight, height, and BMI between patients with early and late water tolerance.

In the present study, a reduced early water tolerance was observed in ten individuals, of whom six (66.7%) demonstrated recovery after six months. In contrast, one patient (5.9%) exhibited early juice reduced tolerance, while none demonstrated late juice reduced tolerance. Despite the significant improvement in water tolerance, early water tolerance was significantly worse than juice tolerance (*p* = 0.0012), while late water tolerance was nearly significantly worse than juice tolerance (*p* = 0.0726).

Patients who experienced challenges with liquid intake following LSG often characterized this issue as a sensation of incomplete emptying of the stomach, often accompanied by discomfort and, in some cases, regurgitation. Likewise, Komaei et al. [14] employed a liquid tolerance questionnaire to assess the quality of liquid intake at one, six, twelve, and 24 postoperative months. During the initial six-month period, still water was identified as the liquid that individuals tolerated most poorly. A significant enhancement in the tolerance to still water was observed after the sixth postoperative month. In addition, Dantas et al. [15] assessed the liquid motion through the water swallowing test. The patients were timed as they consumed 50 ml of water in duplicate, and the number of swallows was documented. They concluded that within two months following the procedure, bariatric surgery may result in more severe changes to liquid bolus swallowing, which then return to normal levels. Also, Elward et al. [7] reported significant early and late differential tolerance between water and juice. Although the improvement in water tolerance at 3 months was insignificant, more than one-third of patients with late difficulty drinking water had late-onset difficulty, suggesting that this differential fluid tolerance may have an intermediate or long-term effect on LSG patients. Because water is a Newtonian fluid and juices are non-Newtonian fluids, Elward et al. [7] hypothesized that the likely perceived difference in tolerance between the two is caused by altered gastric motility following LSG. Newtonian and non-Newtonian fluids are distinguished by their viscosity and fluid behavior in response to shear stress. No matter how much shear is applied at a constant temperature, the viscosity of a Newtonian fluid remains constant [16].

The rheological properties of the fluid have a major influence on both the flow structure and the pressure gradients. In particular, the viscosity of the fluid is exactly proportional to the pressure variations that occur within the antropyloric region [17].

Following LSG, the surgical excision of the gastric pacemaker located at the greater curvature has been proposed as a cause promoting alterations in stomach motility [18, 19]. Because of its importance in initiating and regulating gastric motility, resection of the pacemaker area on the greater curvature of the gastric body during LSG hurts gastric motility in the post-LSG stomach [20].

In the present study, the vertical part of the gastric sleeve was either immotile or exhibited haphazard, insignificant contractions during early and late imaging. Consequently, we sought to study the dynamic MRI patterns in the antropyloric region. Our findings revealed a significant improvement between early and late water and juice MRI contraction patterns. This overall improvement of late MRI motility patterns of water and juice alike revealed a trend, which consisted of reduced antral frequency versus increased in velocity, transit time, and contraction height (Table 3). This motility trend could also be traced among patients with early reduced water tolerance who showed improvement in their late water tolerance (Table 4). Furthermore, that trend was vaguely apparent when comparing water and juice motility patterns, especially in the late phase, yet the contraction height was the only parameter showing highly significant differences in both early and late phases (Table 5).

According to Baumann et al. [9], they used dynamic MRI with 70 frames per minute to describe stomach motility before and after LSG. Following LSG, the stomach appears to be divided into two distinct sections: an accelerated antrum and a predominantly stationary sleeve. The antrum exhibited antral motility in the form of pairwise, quicker, steeper folds at the angular incisure and basal wall. These waves of antral propulsion exhibited an increase in amplitude as they approached the pylorus.

Kwiatek et al. [21] assessed distal antral peristalsis and discovered a periodicity of antral waves between 23 and 40 s, corresponding to frequencies between 1.5 and 2.6 per minute after consuming 500 ml of glucose solution. The speed of the antral propulsive waves (2.7 mm/s) exactly matched the preoperative examination speed. Using a temporal resolution of 70 frames per minute, the fundus showed flat, broad, and slow waves as the primary motility pattern in the stomach before surgery, whereas the antrum showed paired faster and steeper folds at the angular incisure and basal wall. These waves of antral propulsion increased in height as they approached the pylorus and eventually merged to separate the fluid between the fold and the pylorus from the total gastric volume and pump it in that direction.

Our results showed that the %EWL of patients with reduced late water tolerance was 60.90 ± 4.86%, which was lower than that of patients with good late water tolerance, 69.77 ± 9.31%, but without statistically significant differences. Elward et al. [7] reported that the %EWL of patients with early difficult drinking water was 30.5%, which was lower than that of patients with good early water tolerance (33.5%), but the difference was not significant. Patients who had difficulty drinking water reported that they replaced drinking water with larger amounts of juices, especially in the first 6 months after LSG. This may impede weight loss or even lead to weight regain in the long term. It has been posited that adequate hydration may facilitate weight reduction [22]. This substitution, however, has the potential to hinder weight reduction or, over time, result in weight gain. After a period of six months, the study’s participants had achieved a weight reduction of more than fifty percent of their excess weight, with no significant differences observed among them.

### Limitations

The follow-up period was relatively short. Although our sample of 17 patients had a significant prevalence of decreased water tolerance, a larger sample size may help us better understand the extent of the problem. Further studies with extended follow-up are necessary to determine whether water tolerance differs from that of other liquids following LSG. The subjective questionnaire tool we used to assess liquid tolerance was not a validated questionnaire. To the best of our knowledge, there is no validated liquid tolerance questionnaire published yet.

## Conclusions

Time-resolved gastric MRI is a useful tool to explain the post-LSG differential liquid tolerance. The gastric antrum is the only coordinated and functionally motile part of the gastric sleeve. An overall significantly improved motility pattern trend was achieved by the end of the study, with the increase in antral contraction height being the most consistent component of that trend. Water tolerance improves significantly 6 months after LSG but remains worse than juice tolerance. We need more prospective studies with larger sample sizes to predict the normal ranges for different motility patterns, which can help the assessment of cases with post-LSG reduced liquid tolerance.

## Data Availability

No datasets were generated or analysed during the current study.
